# Predicting Perennial Ryegrass Cultivars and the Presence of an *Epichloë* Endophyte in Seeds Using Near-Infrared Spectroscopy (NIRS)

**DOI:** 10.3390/s25041264

**Published:** 2025-02-19

**Authors:** Simone Vassiliadis, Kathryn M. Guthridge, Priyanka Reddy, Emma J. Ludlow, Inoka K. Hettiarachchige, Simone J. Rochfort

**Affiliations:** 1 Agriculture Victoria Research, Bundoora, VIC 3083, Australia; simone.vassiliadis@agriculture.vic.gov.au (S.V.); kathryn.guthridge@agriculture.vic.gov.au (K.M.G.); priyanka.reddy@agriculture.vic.gov.au (P.R.); emma.ludlow@agriculture.vic.gov.au (E.J.L.); inoka.hettiarachchige@agriculture.vic.gov.au (I.K.H.); 2 School of Applied Systems Biology, La Trobe University, Bundoora, VIC 3083, Australia

**Keywords:** *Lolium perenne*, short-term ryegrass, fescue, discriminant analysis, hierarchical model, non-destructive, quality control

## Abstract

Perennial ryegrass is an important temperate grass used for forage and turf worldwide. It forms symbiotic relationships with endophytic fungi (endophytes), conferring pasture persistence and resistance to herbivory. Endophyte performance can be influenced by the host genotype, as well as environmental factors such as seed storage conditions. It is therefore critical to confirm seed quality and purity before a seed is sown. DNA-based methods are often used for quality control purposes. Recently, near-infrared spectroscopy (NIRS) coupled with hyperspectral imaging was used to discriminate perennial ryegrass cultivars and endophyte presence in individual seeds. Here, a NIRS-based analysis of bulk seeds was used to develop models for discriminating perennial ryegrass cultivars (Alto, Maxsyn, Trojan and Bronsyn), each hosting a suite of eight to eleven different endophyte strains. Sub-sampling, six per bag of seed, was employed to minimize misclassification error. Using a nested PLS-DA approach, cultivars were classified with an overall accuracy of 94.1–98.6% of sub-samples, whilst endophyte presence or absence was discriminated with overall accuracies between 77.8% and 96.3% of sub-samples. Hierarchical classification models were developed to discriminate bulked seed samples quickly and easily with minimal misclassifications of cultivars (<8.9% of sub-samples) or endophyte status within each cultivar (<11.3% of sub-samples). In all cases, greater than four of the six sub-samples were correctly classified, indicating that innate variation within a bag of seeds can be overcome using this strategy. These models could benefit turf- and pasture-based industries by providing a tool that is easy, cost effective, and can quickly discriminate seed bulks based on cultivar and endophyte content.

## 1. Introduction

Perennial ryegrass (*Lolium perenne* L.) is an important pasture and turf grass which has high economic impact in temperate zones worldwide. It is the most common pasture grass utilized in Australian and New Zealand agricultural landscapes. The species is favoured due to its digestibility and the ability to form symbiotic relationships with naturally occurring fungal *Epichloë* endophyte species (herein referred to as endophytes). The endophyte enhances pasture performance and persistence, and the production of endophyte-derived alkaloids confers resistance to grazing animals and insect pests [1,2,3,4,5,6,7]. As such, endophytes of perennial ryegrass, as well as the related species Italian ryegrass and tall fescue, have been commercially exploited for the benefit of the pasture and turf industries.

It is important to consider which perennial ryegrass cultivar is sown, as the performance of endophytes can be influenced by the host genotype. The presence and identity of an endophyte, as well as the overall seed quality, are also of key importance, especially when adulterations caused by the mixing of seeds may occur during the breeding, commercialization, and seed production processes. *Epichloë* endophytes associated with perennial ryegrass and tall fescue have been classified into nine different taxonomic groups: *Lp*TG-1 (*Lolium perenne* taxonomic group 1), *Lp*TG-2, *Lp*TG-3, and *Lp*TG-4, *Fa*TG-1 (*Festuca arundinaceum* taxonomic group 1), *Fa*TG-2, *Fa*TG-3, *Fa*TG-4, and *Fa*TG-5 [8,9]. The presence of an endophyte is well known for providing substantial benefits to the host grass, including improved resistance to pest and disease via the production of bioactive compounds including lolitrem B, epxoy-jantirems, ergovaline, peramine, formyl loline, and acetyl loline [10,11]. It is also critical to monitor changes to seed quality traits, including endophyte characteristics, e.g., alkaloid profiles, when considering commercial rights and the development of new cultivars and novel endophytes [6,12,13].

Methods to determine the authenticity and identification of ryegrass cultivars include morphophysiological criteria such as plant breeder rights (PBRs) and DNA-based genotyping [14,15]. Although PBR/PVR registration requires varieties to be morphologically distinct, the uniform and stable (DUS) [16] conduct of field trials and testing for varietal identification or verification [11] based on these criteria may be costly and time-consuming. Pembleton (2016) developed a genotyping-by-sequencing approach for effective discrimination between ryegrass species and the identification of cultivars using bulked samples [14]. Traditionally, endophyte strains are identified using DNA-based [11,15] and genotyping-based techniques that are accurate and robust; however, they require sample destruction and can be expensive for routine use as a quality control tool. Additionally, seeds may have limited availability early in breeding programmes, and only a random subset of seeds can be processed for quality assurance purposes at a given time.

Near-infrared spectroscopy (NIRS) is a fast and non-destructive technique which does not require chemical reagents. The chemical composition of a sample is determined based on the interaction between the surface of the sample and a light source which covers the near-infrared spectral region of the electromagnetic spectrum from around 800 to 2500 nm (equivalent to 12,500–4000 cm^−1^, expressed as wavenumber). NIR light is directed onto the surface of a sample, and a spectrum is provided when the radiation at a specific frequency corresponds to the vibration of a particular chemical bond (e.g., -OH, -CH, -NH, and -SH). These bonds absorb the radiation energy whilst the remaining radiation is reflected or transmitted [17,18]. The water content and other organic compounds, such as proteins, carbohydrates, alcohols, and lipids, are able to be measured [17,18].

The absorption of NIR radiation by organic molecules results in a spectrum which consists of overtone regions. These arise when the energy forces the molecules from a ground state (v = 0) to an excited state (v = 2, the first overtone; or v = 3, the second overtone). The spectrum also contains combination bands, which occur when two or more fundamental vibrations are excited at the same time. This spectral region contains complex wavebands, and hence, individual chemical components are difficult to identify [17,18].

NIRS has wide applications in agricultural settings [19]. For example, the dairy industry utilizes NIRS to evaluate the quality of feed, milk, and dairy products such as cheese and butter [20]. Additionally, soil quality, including soil carbon content, texture and total nitrogen content, can be reliably measured using NIRS methods [21,22], and the technology has been used to develop validation methods for the prediction of forage quality [23,24,25]. The advancement of NIRS instruments, software, and statistical methods has improved the application for many different fields in research, including the quality control of seeds.

It has been over 60 years since the pioneer of NIRS, Karl Norris, used the technology to measure the content of moisture in seeds [26]. Since then, NIRS is routinely used for the assessment of seed quality, vigour, and viability [27], including watermelon [28], soybean [29], pepper [30], and corn [31,32]. NIRS can also be coupled with hyperspectral imaging (HSI) to categorize the quality of grains and seeds [18,33,34]. Such technology is a powerful tool for the identification and classification of seed varieties in cotton [35], barley [36], rice [37,38], maize [39], and wheat [40].

There is also good evidence to suggest that NIRS and NIRS-HSI may be used for the detection of fungal infection in plants and seeds, including *Fusarium* spp. in garlic cloves [41], *Fusarium* spp. in barley and wheat grains [42], *Aspergillus* spp. in rice [43], *Aspergillus* spp. and *Penicillium* spp. in canola seeds [44], and *Aspergillus* spp. in pistachio kernels [45]. In most cases, the damage caused by the fungal infections is visible in the seeds. However, the detection of beneficial endophytic fungi, such as *Epichloë* in perennial ryegrass seeds, is more challenging as mycelia are present within the seed and the external seed appearance is asymptomatic. Nonetheless, research has shown that NIRS and/or NIRS-HIS technology can be used to predict endophyte presence in perennial ryegrass seeds [34], discriminate between different endophyte species [46,47], and also detect and quantify endophyte-produced alkaloids in meadow fescue [48] and perennial ryegrass [49].

The pairing of NIRS data with machine learning algorithms has led to considerable advances in the agricultural industry. Recently, statistical models have been built for classifying seed quality traits [50,51], seed varieties, and endophyte discrimination [34]. Such algorithms aim to capture high classification accuracies. Partial least squares discriminant analysis (PLS-DA), a support vector machine (SVM), and artificial neural network discriminant analysis (ANN-DA) are several algorithm examples which have been applied for the discrimination of single seeds of perennial ryegrass cultivars and *Epichloë* endophyte presence using NIRS-HSI [34]. Here, optimized PLS-DA and ANN-DA models resulted in overall accuracies greater than 89%. These provide promising results for building calibration models. However, for the purpose of seed testing in batches, a greater suite of cultivars and endophyte combinations would be required to build robust models. Additionally, it would be ideal if the developed methods were less laborious than NIRS-HSI, which requires the manual handling of single seeds on Petri dishes or conveyer belts [18,34], whilst maintaining high classification accuracies.

The objective of the current study was to use NIRS coupled with PLS-DA models to classify independently harvested batches of seeds from four genetically distinct perennial ryegrass cultivars [14] and in turn, predict endophyte status using a suite of novel endophyte strains with different alkaloid profiles and representing different taxonomic groups that form natural or artificial associations with perennial ryegrass [8,11,14,15].

The methods were designed to be useful for a novice with no expert training. It was also designed to be fast and simple, for example, by using a single scoop of seeds and by using optimized classification models to build a decision tree using a hierarchical model builder, whereby ‘test’ samples may be ‘dropped’ into the model. The aim of this application was to create a robust method for testing seeds for quality assurance purposes throughout the breeding, seed production, and distribution processes.

## 2. Materials and Methods

### 2.1. Sample Preparation

Perennial ryegrass seeds were supplied by Barenbrug, Christchurch, New Zealand (Table 1). A total number (n) of 135 individual bags of seeds were collected across a range of different harvest years and locations. The dataset consisted of four cultivars: Alto (n = 36; date range from 5 July 2005 and 11 July 2018), Bronsyn (n = 32; date range from 30 June 2005 and 11 July 2018), Maxsyn (n = 10; date range from 11 July 2018 and 1 September 2017), and Trojan (n = 57; date range from 6 March 2013 and 23 September 2019). The cultivars were host to a suite of twelve different *Epichloë* spp. endophyte strains including AR1, AR37, NEA2/6, NEA10, NEA11, NEA12, NEA2, NEA21, NEA23, NEA3, NEA6, and SE. Endophyte presence and identity was confirmed via Kompetitive Allele-Specific PCR (KASP) analysis [11]. The total number of bags of seeds with an endophyte present (E+) was n = 119. Each cultivar also had representatives of seeds without an endophyte, and we henceforth referred to these instances as endophyte absent (E−). The total number of E− seeds was n = 16.

Approximately 10 g of seeds were sub-sampled into 50 mL tubes (Cellstar, Greiner Bio-One, Kremsmünster, Austria) and stored in the dark within a controlled environment room (CER) maintained at 4 °C.

### 2.2. NIRS Data Acquisition

The NIR spectra of perennial ryegrass seeds were obtained using an FT-NIR spectrometer (MPA II-Multi Purpose FT-NIR Analyzer, Bruker Optic, Inc., Billerica, MA, USA). The system measured diffuse reflectance over a wavelength range of 11,536–3952 cm^−1^ (equivalent to 867–2530 nm). A total of 949 data points were collected at a resolution of 16 cm^−1^, and 64 scans were measured within a measurement period of 25 s.

A 50 mm integrating sphere cup with a glass bottom (Bruker Optic, Inc., Billerica, MA, USA) was filled with seeds (approximately 10 g), ensuring that the bottom was completely covered. To ensure high reproducibility of the heterogenous samples, the cup was placed in a sample rotator, and each sample was scanned six times. The seed were gently agitated between each scan, thereby providing suitable replication of sub-samples required to build calibration models. The background was automatically recorded every hour using a gold-coated reference (64 scans, 25 s). A total of 810 scans were acquired using the OPUS 8.2.21 software (Bruker Optic, Inc., Billerica, MA, USA). The resulting spectra displayed the measure of absorbance.

### 2.3. NIRS Data Pre-Processing

OPUS datafiles were imported into MATLAB v.2022a (Mathworks, Natick, MA, USA) coupled with PLS_Toolbox v. 9.0 (Eigenvector Research Inc., Manson, WA, USA) for data analysis. The raw spectra were inspected, and class information, including cultivar identification (Alto, Bronsyn, Maxsyn or Trojan) and endophyte status (presence, E+ or absence, E−), was assigned. Data were randomly organized and then trimmed from 9000 to 3952 cm^−1^ (1111–2530 nm), resulting in a total of 632 data points.

The raw spectra were corrected by applying several pre-processing techniques aimed at removing systemic noise due to light scattering, variation in particle size, and instrumental drift. The removal of such physical interferences allowed for the optimization of models by improving signal to noise. The pre-processing techniques included the correction of the trend (Detrend) and scatter correction via mean multiplicative signal/scatter correction (MSC), standard normal variate (SNV), and extended multiplicative scatter/signal correction (EMSC). Smoothing and spectral derivatives were addressed by applying the Savitzky–Golay first and second derivatives with polynomial order 2 and 15 points in the smoothing window (SavGol-1 and SavGol-2, respectively). Scaling and centering were corrected via the application of mean centering. Eleven pre-processing parameters were applied to raw spectra using a combination of the treatments.

### 2.4. PLS-DA and Model Evaluation

PLS-DA models were used to optimize the separation of classes (cultivar or endophyte status) by coupling the NIR spectral data in the X-block to the categorical data in the Y-block using a latent variable approach. Cross validation (CV) was performed using Venetian blinds with 10 data slits and one sample per blind. For the prediction of each class, the data were split into a 75% calibration (training) dataset and a 25% validation (test) dataset using the Kennard-Stone algorithm in PLS_Toolbox. This method ensures that a uniform selection of samples is represented in both the calibration and validation datasets. It achieves this by using a stepwise procedure in which samples are selected far from the already selected samples (the Euclidian distance) [52].

PLS-DA models were first used to perform binary classification models for the discrimination of individual cultivars (i.e., Alto vs. Bronsyn vs. Maxsyn vs. Trojan) and endophyte presence, irrespective of cultivar (i.e., E+ vs. E−). Models were evaluated based on the classification error (Class. Err.) using the following equation:

                                              Class Err.=average of false positive rate and false negative rate for class,=1−(sensitivity+specificity)/2.

Optimal PLS-DA models were determined by overall accuracy in which the class errors of prediction (Class. Error. Pred) and/or classification errors via cross validation (Class. Error. CV) were lowest for each class. The details for model evaluation were followed according to Reddy et al. [34].

### 2.5. Nested PLS-DA Models and Development of a Hierarchial Classification Model

The PLS-DA models were used to develop a hierarchical classification model using the hierarchical model builder (HMB) in PLS_Toolbox v. 9.0 (Eigenvector Research Inc., Manson, WA, USA). Often, classification by PLS-DA can be compromised when the number of variables or classes is high. To break down the complicated classification problems, separate simplified models were created by combining different classes and subsequently modelling the individual classes of that combination using a hierarchical approach.

The first hierarchical model aimed to automatically classify the seeds of perennial ryegrass cultivars. Each node of the model was determined via the classification obtained by the most suitable PLS-DA model (calibration dataset). The pipeline was broken down into several ‘steps’, which related to separate classification rules.

A similar approach was developed for the classification of endophyte status. Here, four separate hierarchical models were developed to classify the presence of endophyte status (E+ or E−) within Alto, Bronsyn, Maxsyn, and Trojan, respectively.

**Figure 1:**
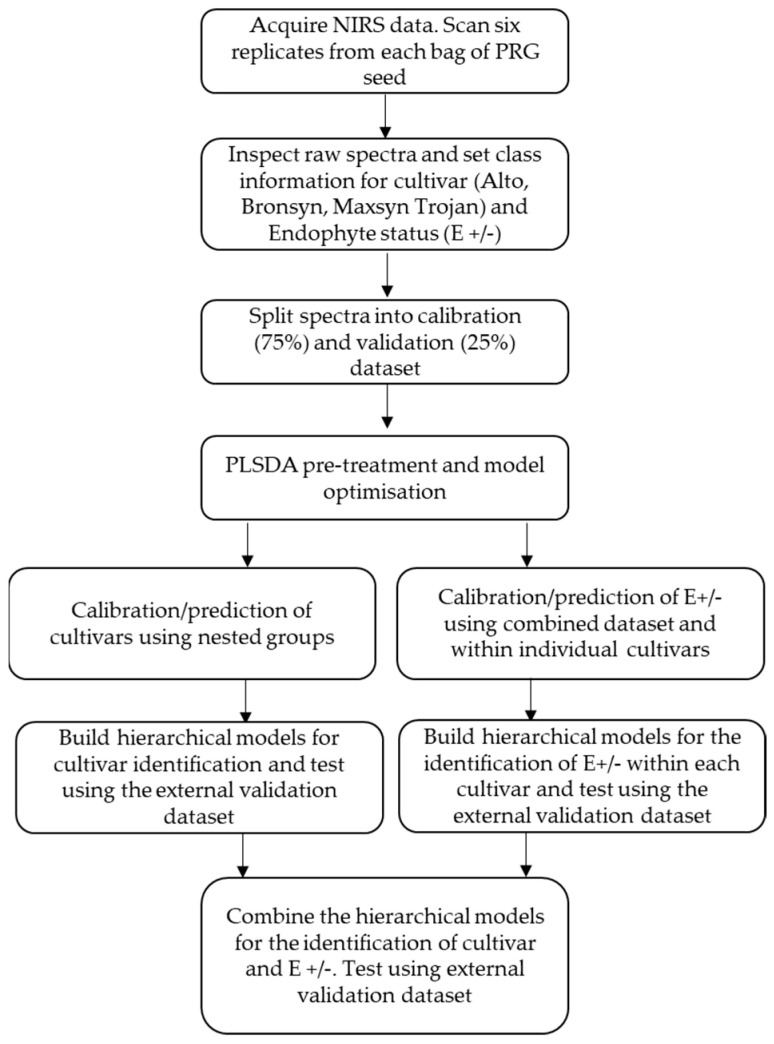
Flow chart illustrating the steps taken to acquire, process, and analyze perennial ryegrass (PRG) seeds for the calibration and prediction of cultivars (Alto, Bronsyn, Maxsyn, and Trojan) as well as endophyte presence or absence (E+/−).

To test the performance of the hierarchical models, the validation (test) spectra were loaded, and resulting ‘hits’ or ‘misses’ (i.e., correct classification or misclassifications) were described via text output. Finally, the five individual hierarchical models were combined. This allowed for the determination of all cultivars to be identified alongside endophyte presence using a single model. Again, total performance was assessed by testing the full validation dataset. Modelling steps were combined, and the pipeline of analysis is shown in Figure 1.

### 2.6. Pipeline

A flowchart summarizing the methods is indicated in Figure 1.

## 3. Results and Discussion

### 3.1. Spectral Overview and Pre-Processing

**Figure 2:**
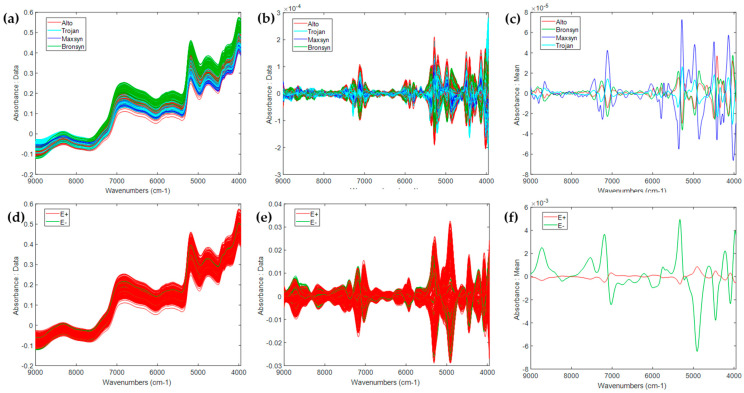
The top figures show the raw NIR spectra: (**a**) spectra treated with Detrend, MSC-mean, SavGol-2, and mean centering (**b**) as well as the mean pre-processed NIR spectra (**c**) of seed classified by cultivar (Alto, red line, Trojan pale blue, Maxyn dark blue and Bronsyn green). The bottom figures show the raw NIR spectra: (**d**) spectra treated with Detrend, SNV, SavGol-1, and mean centre (**e**) as well as the mean pre-processed NIR spectra (**f**) of seed classified by endophyte presence (E+, red line) or absence (E−, green line). The variables represent 632 data points over a spectral range of 9000–3952 cm^−1^.

The raw NIR spectra obtained from the measurement of perennial ryegrass seed samples are illustrated in Figure 2a,d. Data were classed according to cultivar and endophyte presence. The ‘peaks and valleys’ indicate differences in the chemical constituents of the ryegrass samples, and the spectra resemble those of other chemically complex agricultural products including chickpea seeds, rice, and endophyte-infected meadow fescue [48,53,54].

The initial inspection of the raw NIRS data revealed no visual anomalies resulting from poor spectra. Initially, 810 sub-sample spectra (scans) were obtained, but three outliers were detected during the pre-treatment of raw spectra, according to unusual Q-residuals. The outliers consisted of a single scan of Alto-NEA2, as well as two scans of Alto-WE. These were removed from the dataset, thereby reducing the number to 807 scans. As summarized on Table 1, Trojan represented the largest dataset (n = 342 scans), followed by Alto (n = 213 scans) and Bronsyn (n = 192 scans). The smallest dataset consisted of Maxsyn seeds (n = 60 scans). Endophytes were present in most of the seed samples (n = 713 scans) compared to those without an endophyte (n = 94 scans).

When analyzing solid samples, undesirable spectral variations are caused by the scattering of light and differences in the path of light. This results in major variations in the dataset, often seen as baseline shifts and other non-linearities [55]. The NIR spectra must be corrected to remove any artefacts or physical interferences which may interfere with subsequent modelling. The simplest approach to remove additive noise from NIR spectra involves mean centering. This method simply calculates the mean of each variable before subtracting it [51]. Additionally, detrending SNV, MSC, and EMSC, as well as their derivatives, are alternative methods that are commonly used to pre-treat raw NIR spectra [51,55].

Detrending removes tilted baseline variation by subtracting the linear or polynomial fit of a baseline from the original spectrum [56]. MSC and SNV are the most widely used techniques which are applied for scatter correction. MSC aims to regress a measured spectrum against a reference spectrum and then corrects the measured spectrum using the slope of this linear fit [56,57]. SNV is like MSC, but a reference spectrum is not required. SNV calculates the mean and variance; then, it subtracts the mean and divides it by the variance [51]. EMSC is an extension to MSC whereby noise is removed by performing polynomial fitting to the reference spectrum, followed by the correction of the baseline on the wavelength axis [55]. Derivatives are applied to remove additive and multiplicative effects in the spectra [55]. An application of the Savitzky–Golay filter removes high frequency noise, and hence, the NIR signal is smoothed. The amount of smoothing can be modified by adjusting the window size and the number of polynomials and is often used in samples with different particle sizes [51]. The first derivative removes the baseline whilst the second removes the baseline alongside the linear trend [55].

It is important to evaluate the impact of different pre-processing parameters on raw data for the purpose of model optimization, and indeed, two different methods were employed for the discrimination of cultivar and endophyte presence (Supplementary Table S1). Figure 2b,c,e,f show the spectra after the optimal preprocessing techniques had been applied. The mean spectra (Figure 2c,f) showed differences between the distinct classes for cultivar and endophyte presence, respectively, according to specific regions within the NIR spectrum.

The application of the pre-processing steps reduced variations in the y-axis and absorption bands in the region between approximately 6896 and 5184 cm^−1^ (1450–1930 nm) which are related to the moisture (-OH stretch in the first overtone) [24]. The absorption bands are related to critical functional groups such as carbon and hydrogen atoms (C-H), oxygen and hydrogen atoms (O-H), and ammonia and hydrogen atoms (N-H) [24,58]. In general, the main absorption peaks were located at wavelengths at approximately 8230 cm^−1^ (1202 nm) corresponding with C-H in the second overtone, 6824 cm^−1^ (1465 nm) corresponding with N-H stretch in the first overtone, 5736 cm^−1^ (1740 nm) corresponding to S-H stretch in the first overtone, 5176 cm^−1^ (1931 nm) corresponding to a combination of O-H stretch/HOH deformation, 4776 cm^−1^ (2094 nm) corresponding to the C-H combination, and 4304 cm^−1^ (2323 nm) corresponding to a combination of C-H stretch/CH2 deformation [58].

### 3.2. Discrimination of Perennial Ryegrass Cultivars

#### 3.2.1. PLS-DA Models for Cultivar Determination

The impact of different spectral pre-treatment parameters was firstly evaluated by performing PLS-DA models for the cultivars (i.e., Alto vs. Bronsyn vs. Maxsyn vs. Trojan). The result for calibration, cross validation, and prediction models aimed to discriminate between the individual cultivars (Supplementary Table S2). The optimal model was selected based on model predictive performance as well as overall accuracy as described by Reddy et al. [34]. A combination of Detrend, MSC-mean, SavGol-2 and mean centre (Supplementary Table S2, Model 5) provided the lowest class error of prediction (CEP) for the individual cultivars (Alto, 3.0%; Bronsyn, 11.0%; Maxsyn, 1.0% and Trojan, 9%) as well as the lowest cross validation (CV) error (Alto, 2.0%, Bronsyn, 11.0%; Maxsyn, 1.0% and Trojan, 13%). The model was performed with eight latent variables (LVs), and the overall accuracy was greater than 87%.

Similar results for cultivar identification using NIR-based technologies have been described in the literature. Zhu et al. employed PLS-DA models to discriminate between eleven varieties of cotton seeds, resulting in a prediction accuracy of 80.4% [35]. The PLS-DA models performed by Kong et al. also resulted in a classification accuracy of prediction greater than 80% when discriminating between four cultivars of rice seeds [38]. Moreover, Reddy et al. [34] employed NIR-HSI to build models aimed at predicting five perennial ryegrass seed cultivars. Here, several modelling techniques were applied, and overall accuracies were determined at 90% using ANN-DA models, compared to 89.2% using PLS-DA models.

In the current study, the optimized parameters were used for subsequent PLS-DA models using nested class groups (Table 2). The purpose of nesting the classes was to create a model that can simultaneously predict both positive outcomes (e.g., belonging to class 1) and negative outcomes (e.g., belonging to class 2, which in this case was all the other cultivars) [59]. The steps in which the nested models were performed were determined based on the CEP and cross validated results stated above.

The overall accuracies were improved using this nested group approach. Maxsyn, which was modelled first against all other cultivars (Step 1), was classified with a CEP of 1.9% and a CV error of 3.8%. The overall accuracy was 96.2% using three LVs. Maxsyn was thereby removed from the modelling pipeline. Alto was then modelled against Bronsyn and Trojan (Step 2) and classified with a CEP of 1.1% and a CV error of 1.4%. The overall accuracy was 98.6% using seven LVs. Alto was subsequently removed from the modelling pipeline. The final model aimed to discriminate Bronsyn from Trojan (Step 3), resulting in a CEP of 4.4% and a CV error of 5.9%. The overall accuracy was 94.1% using six LVs.

**Figure 3:**
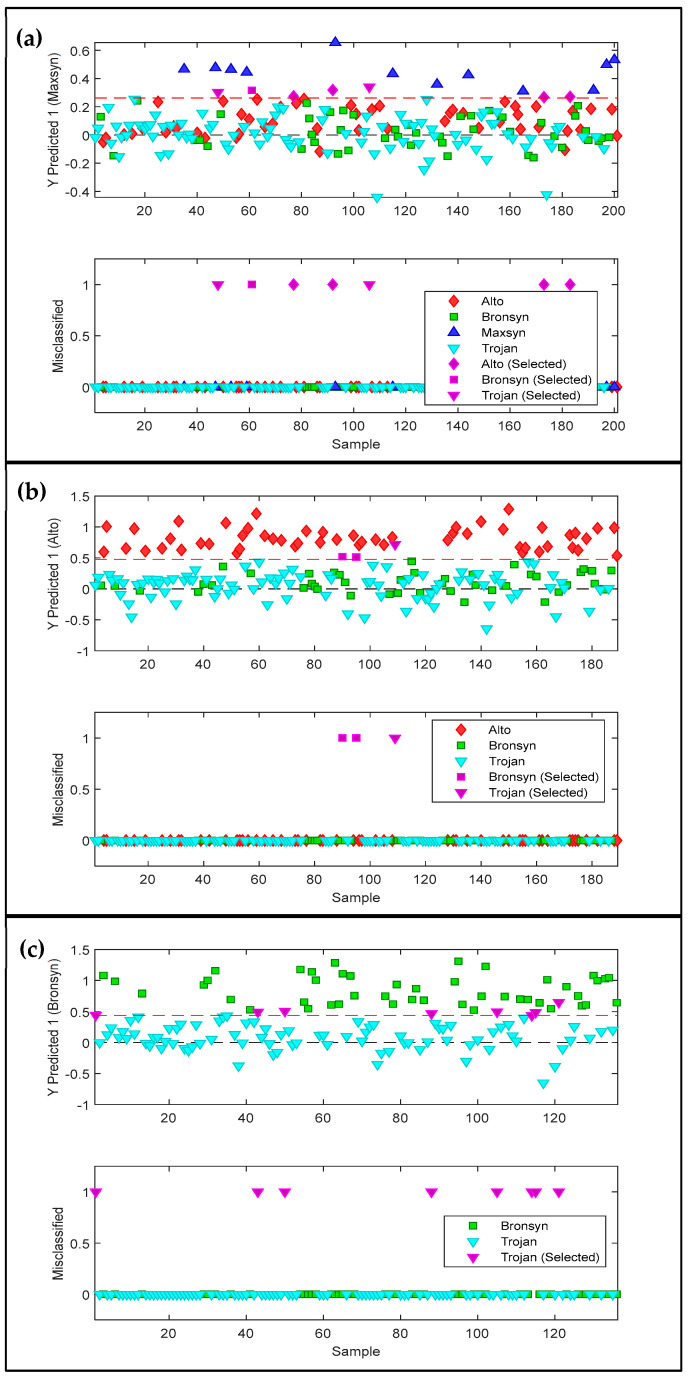
PLS-DA sample/score plots (**top** figures) and the misclassified sub-samples (**bottom** figures) shown for the prediction of (**a**) Maxsyn from Alto, Bronsyn, and Trojan (Step 1); (**b**) Alto from Bronsyn and Trojan (Step 2); and (**c**) Bronsyn from Trojan (Step 3). The misclassified sub-samples are shown in purple. Data show the result of the validation (test) dataset: Alto (n = 53), Bronsyn (n = 46), Maxsyn (n = 12), and Trojan (n = 90). The red dotted line is the line of discrimination.

The models show that at each classification step, the sensitivity (prediction) was 100%, correctly identifying all samples belonging to class 1. However, specificity errors were obtained from misclassifications belonging to class 2. These misclassified sub-samples/scans are depicted in Figure 3. Here, the corresponding sample/score plots which are predicted as class 1 for each step in the modelling pipeline appear above the line of discrimination.

#### 3.2.2. Hierarchical Classification and Model Validation for Cultivar Discrimination

It is common to use PLS-DA models for classification purposes, but models tend to fail when there are many classes to separate [60]. Singh et al. employed NIR-HSI coupled with PLS-DA models to discriminate between 35 types of barley varieties; however, the accuracy of predictions from the PLS-DA models decreased as the number of varieties increased [36]. Deep neural networks may be used to overcome this problem, but many samples are required, and this may be problematic when trying to classify seeds due to costs and availability. An alternative is to build automated hierarchical models, or decision trees, which can ‘break down’ big classification problems into smaller ones [60].

**Figure 4:**
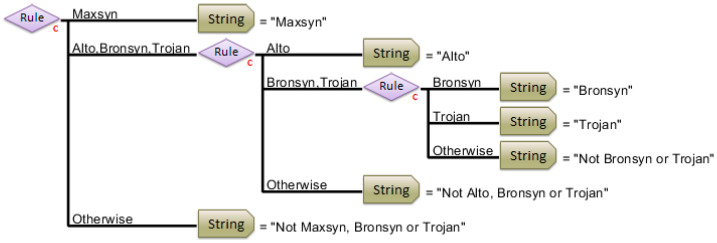
Hierarchical classification model (image extracted from PLS_Toolbox) for the discrimination of perennial ryegrass cultivars. Each rule is determined via the classification models (small c), as illustrated in Table 2 (steps 1–3). The first rule (Maxsyn vs. Alto, Bronsyn, and Trojan) utilizes Step 1 in the modelling pathway. The second rule utilizes Step 2 (Alto vs. Bronsyn or Trojan), and the third rule utilizes Step 3 (Bronsyn vs. Trojan). The strings determine the identity of the cultivar, based on the model output. The ‘otherwise’ function determines an error, or fail, in the associated string of the model.

The hierarchical classification model successfully discriminated the seed samples according to their respective cultivar class (Figure 4). Each rule was determined using the classification models, as illustrated in Table 2 (Steps 1–3). The first rule (Maxsyn vs. Alto, Bronsyn, and Trojan) utilizes Step 1 in the modelling pathway. The second rule utilizes Step 2 (Alto vs. Bronsyn or Trojan), and the third rule utilizes Step 3 (Bronsyn vs. Trojan). The decision tree was tested by simply ‘dragging and dropping’ the validation dataset onto the HMB. The returned results of cultivar classification were immediate, and the number of misclassified sub-samples is illustrated in Table 3.

At the first step of the classification stage, 3.7% of the sub-samples from Alto (four out of fifty-three), Bronsyn (one out of forty-six), and Trojan (two out of ninety) were misclassified as Maxsyn. At the second step, 2.2% of the sub-samples from Bronsyn (two out of forty-six) and Trojan (one out of ninety) were misclassified as Alto. At the third classification step, 8.9% of the Trojan sub-samples were misclassified as Bronsyn (eight out of ninety). Results indicate the importance of sub-sampling. Even though some sub-samples were misclassified, in all instances, 4–5 out of 6 were indeed correctly classified, thus highlighting the method as a powerful tool.

### 3.3. Discrimination of Endophyte Status

#### 3.3.1. PLS-DA Models for Endophyte Determination

There is good evidence to suggest that NIRS is a useful tool which may be used for the detection of fungal infection in grains [42,43,44]. Lim et al., used NIRS coupled with PLS-DA models to discriminate between grain (hulled barley, naked barley, and wheat) samples contaminated with *Fusarium* spp., resulting in classification rates greater than 98% [42]. However, the detection of endophytes in seeds is more difficult as the endophyte’s presence is asymptomatic. This is because the endophyte is located within the seed, whereas disease-causing fungal pathogens are generally external.

The optimal pre-processing parameter was determined for the discrimination of endophyte status, irrespective of cultivar. A combination of Detrend, SNV, SavGol-1, and mean centre (Supplementary Table S3, Model 6) provided the lowest CEP (12.3%) and lowest CV error (14.6%). The model was performed with nine LVs, and the overall accuracy was 85.4%. This result was slightly lower than that reported by Reddy et al., in which PLS-DA models resulted in the classification of endophyte presence with an overall accuracy of 89% in perennial ryegrass seeds [34]. According to Reddy et al., only one endophyte strain was used for testing endophyte status, whilst twelve strains representing four different *Epichloë* species and six different qualitative alkaloid profiles (Table 1) were employed in the current study. The slight difference in the accuracy results may therefore be due to greater endophyte variability. The better performance may also be due to how the data were acquired. Compared to NIRS which results in a single spectrum, NIRS-HSI allows spectra to be collected for each pixel from the averaged sample image, thus providing spatial distribution and chemical composition information [33,61]. While both utilize NIRS technology, the methods for cultivar and endophyte discrimination described here using NIRS alone differ in their purpose to the NIRS-HSI described by Reddy [18], i.e., rapid batch scale analysis versus single seed analysis for identification and selection.

The predictive errors and overall accuracies were generally improved by utilizing models aimed at classifying E+ and E− within each cultivar class (Table 4). For all models, the sensitivity (prediction) was greater than 88%. The overall accuracy for E+ and E− in Maxsyn was 96.3% (CEP, 0.0%; CV error, 3.7%). In Bronsyn seeds, the overall accuracy was 90.2% (CEP, 9.8%; CV error, 9.8%). The models did not perform as well for Trojan and Alto seeds. In Trojan, the overall accuracy was 86.3% (CEP, 11.8%; CV error, 13.7%), whilst an overall lower accuracy of 77.8% was determined for Alto seeds (CEP 6.0%, but the CV error was 22.2%).

**Figure 5:**
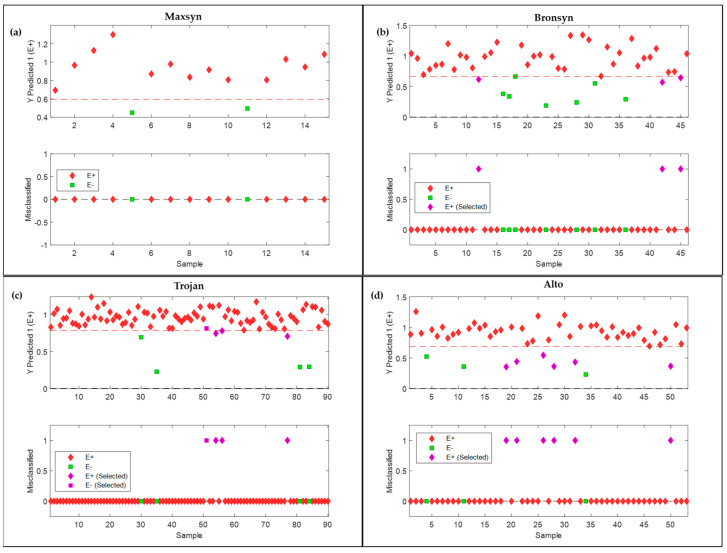
PLS-DA samples/scores plots (**top** figures) and the misclassified sub-samples (B figures) shown for the prediction of endophyte presence (E+ or E−) in (**a**) Maxsyn, (**b**) Bronsyn, (**c**) Trojan and (**d**) Alto. The misclassified sub-samples are shown in purple. Data shows the result of the validation (test) dataset: Maxsyn (E+, n = 13 and E−, n = 2), Alto (E+, n = 50 and E−, n = 3), Bronsyn (E+, n = 39 and E−, n = 7) and Trojan (E+, n = 233 and E−, n = 23). Red dotted line is the line of discrimination.

Figure 5 shows the corresponding sample/score plots for the prediction of endophyte status in each respective batch of cultivar seeds, with misclassified sub-samples highlighted.

#### 3.3.2. Hierarchical Classification and Model Validation for Endophyte Discrimination

For endophyte discrimination, four separate hierarchical classification models were constructed, each in respect to their cultivar classes. The number and type of misclassified sub-samples are illustrated in Table 5. No sub-samples were misclassified in the Maxsyn seed batches. However, 6.5% of sub-samples were misclassified in Bronsyn, 4.4% of sub-samples were misclassified in the Trojan seed batches and 11.3% of sub-samples were misclassified in the Alto seed batches.

### 3.4. Combining Hierarchial Models for Fast Determination of Cultivars and Endophyte Status

It is common practice to develop hierarchical classification models based on PLS-DA models. Such models have been utilized in many types of industries, for example, the classification of raw materials for the tyre industry [62], for the classification of blood stains [63], and for the discrimination of wine vinegars [64]. Marchi et al. discussed the benefits of employing hierarchical classification models to break down an array of larger datasets consisting of sugar, chickpeas, potatoes, and fish [60]. Here, the hierarchical classification models outperformed classic PLS-DA models, especially when the datasets were large.

This study shows that hierarchical models can be used to discriminate between perennial ryegrass cultivars and endophyte status, providing a rapid and inexpensive means to determine the identity of an unknown sample, provided that there are reference samples for comparison. The methods developed allow users to check the error at each individual step of the pipeline.

**Figure 6:**
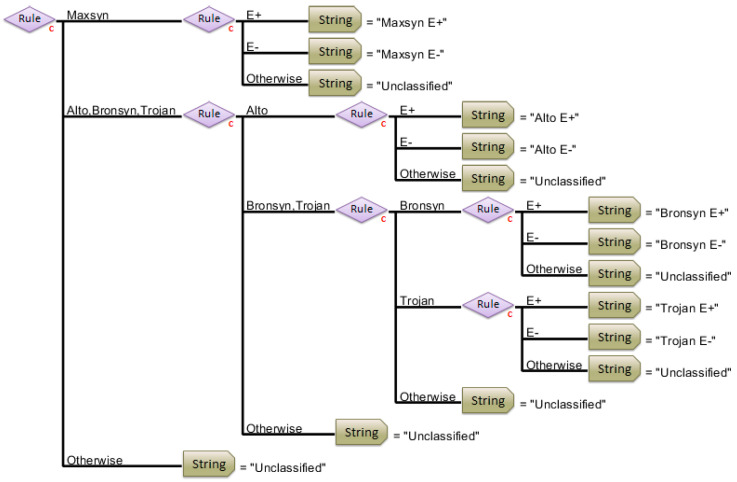
Hierarchical classification model for the discrimination of perennial ryegrass cultivars and the presence or absence of an endophyte (E+ or E−). The image is extracted from PLS_Toolbox.

However, there are further advantages to confirming the cultivar and endophyte status from seed batches within a single step. The larger hierarchical model (Figure 6) resulted in the successful classification of the dataset. There was a slight increase in the number of total misclassifications (13% of sub-samples were misclassified irrespective of cultivar or endophyte status) (Table 6). However, regarding cultivars, only 4.5% of the sub-samples (9 out of 201) were misclassified, and similarly, only 6% of the sub-samples (12 out of 201) were misclassified according to E+ or E−. Six sub-samples (3%) were misclassified according to both cultivar and endophyte presence, which included one which was not able to be classified.

This study demonstrates how NIR coupled with PLS-DA models and subsequent automated hierarchical classification models may be used for the fast discrimination of seed samples, irrespective of harvest year, location, the number of years in cold storage, or the strain of the endophyte. The pipeline for creating the hierarchical classification models was followed according to Marchi et al. [60]. For future modelling, it is recommended that binary classification models should be performed for each class versus each class (i.e., cultivar vs. cultivar or E+ versus E−). The classes which result in the lowest classification error can be merged into a single class group and then analyzed until only two classes remain.

The current study identified a small percentage of misclassified sub-samples, which were used to describe replicated scans following sample mixing from the same original bag of seeds. The purpose of scanning seeds multiple times was three-fold: (1) to build the calibration and validation dataset required for robust modelling, (2) to account for variation in seeds within bags and/or batches, and (3) to account for any variation that may occur when scanning the sample. This strategy is also important for building seed reference collections that will be used in future seed-testing facilities. There were no obvious spectral indications as to why some sub-samples/scans were misclassified by the model. However, it is clear that some sub-samples may yield different classifications. Therefore, it is advised that sub-sampling is included in the future development of classification models, as high overall classification reliability can be achieved. In this study, we set a threshold for classification purposes; for example, if four out of six seeds are correctly classified, then one might assume all are correctly identified.

The validity of cross validation and prediction models depends on the dataset. Small sample numbers may result in overfitting, whilst larger datasets with fewer representatives may result in poor accuracies. For future modelling, it would be desirable to obtain a greater number of seeds with representative samples, for example, a greater number of seeds without endophytes or a greater number of seeds with the same endophyte profile. In real-world scenarios, seed suppliers and seed companies would require robust models which represent a variable dataset with many samples for each class. This would enable robust models in which seed quality (related to endophyte presence) and seed identity may be confirmed with greater accuracy.

## 4. Conclusions

NIRS is a fast, non-destructive technology that can be used for the highly accurate determination of cultivar and endophyte status of ryegrass seeds. The analysis of seeds using the method described here has the further advantage of requiring little expert training, enabling the rapid and accurate discrimination of additional new samples and the use of the technology for routine analysis.

Seeds could be correctly classified irrespective of harvest year, harvest location, the number of years in cold storage, as well as strain of the resident endophyte, thus enabling the easy development of a database for each trait, cultivar, and endophyte status, as investigated in this study. Once a sufficient database of samples is established, this technology could be applied at any stage of the cultivar/endophyte development process, from pre-breeding and breeding to seed production and seed storage.

The objective of our study was to not only make robust models aimed at using seeds to discriminate cultivar and endophyte status but also to build a hierarchical classification modelling tool that can be utilized for the routine analysis of new samples. The use of nested PLS-DA allowed for cultivar and endophyte status to be classified with high accuracy. The development and use of the hierarchical classification models allowed for the immediate determination of cultivar and endophyte status of the seeds, with only a small percentage of misclassified sub-samples observed. The use of sub-samples further improved classification as both cultivars and endophytes were correctly determined for all individual bags of seeds.

The regular testing of seeds, in particular endophyte status, is important throughout the breeding, seed production, and seed distribution processes, thereby ensuring end-users receive a quality product. Our study presents a promising NIRS-based method that may be utilized more broadly, for example, across a larger number of commercially relevant cultivars or extended to other forage and turf species including but not limited to annual ryegrass and tall fescue. The implementation of such methods would benefit the turf and pasture industry by providing a tool to screen seeds that is easy, fast, cost effective, and can accurately discriminate seeds based upon cultivar and endophyte content.

## Figures and Tables

**Table 1:** Perennial ryegrass seed samples used for the acquisition of NIRS data. Four cultivars (Alto, Bronsyn, Maxsyn, and Trojan) hosting a suite of twelve endophytes (endophyte present, E+), were analyzed alongside those without an endophyte, WE (endophyte absent, E−).

Endophyte Status	Endophyte Strain	Taxonomic Group	Alkaloid PROFILE	Alto	Bronsyn	Maxsyn	Trojan	Total ^2^
E+	AR1	*Lp*TG-1	P	2 (12)	2 (12)	NA	2 (12)	6 (36)
E+	NEA2/6	*Lp*TG-1	L^low^EP	2 (12)	1 (6)	NA	28 (168)	31 (186)
E+	NEA10	*Lp*TG-1	EP	3 (18)	3 (18)	1 (6)	3 (18)	10 (60)
E+	NEA2	*Lp*TG-1	L^low^EP	4 (24) ^3^	2 (12)	1 (6)	3 (18)	10 (60)
E+	NEA3	*Lp*TG-1	EP	3 (18)	2 (12)	1 (6)	3 (18)	9 (54)
E+	NEA6	*Lp*TG-1	EP	4 (24)	4 (24)	NA	3 (18)	11 (66)
E+	SE	*Lp*TG-1	LEP	4 (24)	4 (24)	1 (6)	NA	9 (54)
E+	NEA11	*Lp*TG-2	EP	3 (18)	3 (18)	1 (6)	3 (18)	10 (60)
E+	AR37	*Lp*TG-3	J	3 (18)	2 (12)	NA	2 (12)	7 (42)
E+	NEA12	*Lp*TG-3	J	2 (12)	NA	2 (12)	2 (12)	6 (36)
E+	NEA21	*Fa*TG-3	LolP	NA	2 (12)	1 (6)	2 (12)	5 (30)
E+	NEA23	*Fa*TG-3	LolP	NA	2 (12)	1 (6)	2 (12)	5 (30)
E−	WE	NA	NA	6 (36) ^4^	5 (30)	1 (6)	4 (24)	16 (96)
Total ^1^				36 (216)	32 (192)	10 (60)	57 (342)	135 (810)

Individual bags of seeds were scanned six times each (sub-samples, indicated in parenthesis). Totals are shown for ^1^ cultivars and ^2^ endophytes. Three outlier scans were later removed from the dataset, including ^3^ Alto NEA2 (one scan) and ^4^ Alto WE (two scans). *Lp*TG, the *Lolium perenne* taxonomic group. *Fa*TG, the *Festuca arundinacea* taxonomic group. NA, not applicable. L, lolitrem B. E, ergovaline. P, peramine. J, epoxy-janthitrems. Lol, loline.

**Table 2:** PLS-DA calibration (Cal), cross validation (CV) and prediction (Pred) models for the discrimination of perennial ryegrass seed cultivars using nested class groups. Misclassification rates vary from 1.1 to 4.4%.

Step	LVs	Cultivar	Sensitivity (Cal)	Class Error (Cal)	Sensitivity (CV)	Class Error (CV)	Sensitivity (Pred)	Class Error (Pred)
1	3	Maxsyn	0.98	3.6%	0.98	3.8%	1.00	1.9%
		Alto, Bronsyn, Trojan	0.95	3.6%	0.94	3.8%	0.96	1.9%
2	7	Alto	0.99	0.9%	0.98	1.4%	1.00	1.1%
		Bronsyn, Trojan	1.00	0.9%	0.99	1.4%	0.98	1.1%
3	6	Bronsyn	0.94	4.3%	0.93	5.9%	1.00	4.4%
		Trojan	0.98	4.3%	0.96	5.9%	0.91	4.4%

LVs, latent variables. Steps 1–3 indicate the pathway to the prediction of individual cultivars. The calibration dataset included Alto (n = 160), Bronsyn (n = 146), Maxsyn (n = 48), and Trojan (n = 252). The validation dataset included Alto (n = 53), Bronsyn (n = 46), Maxsyn (n = 12), and Trojan (n = 90).

**Table 3:** The number of misclassified sub-samples identified using the hierarchal model which was built to discriminate between cultivars of perennial ryegrass seeds. Misclassification rates vary from 2.2 to 8.9%.

Step (Rule)	Cultivar (Classification)	Cultivar (Actual)	No. of Times Misclassified	Total Misclassified (per Cultivar)	Total Misclassified (per Step/Rule)
1	Maxsyn	Alto	4 of 53	7.5%	
		Bronsyn	1 of 46	2.2%	
		Trojan	2 of 90	2.2%	3.7%
2	Alto	Bronsyn	2 of 46	4.3%	
		Trojan	1 of 90	1.1%	2.2%
3	Bronsyn	Trojan	8 of 90	8.9%	8.9%

The validation dataset was used to test the hierarchical model. The number of NIRS sub-samples (1 scan/sub-sample) included Alto (n = 53), Bronsyn (n = 46), Maxsyn (n = 12), and Trojan (n = 90).

**Table 4:** PLS-DA calibration (Cal), cross validation (CV) and prediction (Pred) models for the discrimination of endophyte presence (E+) or absence (E−) in cultivars of perennial ryegrass seeds. Misclassification rates vary from 0 to 11.8%.

Model	LVs	Endophyte Status	Sensitivity (Cal)	Class Error (Cal)	Sensitivity (CV)	Class Error (CV)	Sensitivity (Pred)	Class Error (Pred)
Maxsyn ^1^	3	E+	0.98	1.2%	0.93	3.7%	1.00	0.0%
		E−	1.00	1.2%	1.00	3.7%	1.00	0.0%
Bronsyn	6	E+	0.95	9.0%	0.94	9.8%	0.92	3.8%
		E−	0.87	9.0%	0.87	9.8%	1.00	3.8%
Trojan	5	E+	0.91	4.7%	0.88	13.7%	0.97	11.8%
		E−	1.00	4.7%	0.84	13.7%	0.80	11.8%
Alto	4	E+	0.85	12.6%	0.81	22.2%	0.88	6.0%
		E−	0.90	12.6%	0.74	22.2%	1.00	6.0%

^1^ A new calibration/validation dataset was created for Maxsyn as the first did not include any E− sub-samples in the validation set. LV, latent variables. The calibration dataset included: Maxsyn (E+, n = 41 and E−, n = 4), Alto (E+, n = 129 and E−, n = 31), Bronsyn (E+, n = 123 and E−, n = 23) and Trojan (E+, n = 233 and E−, n = 19). The validation dataset included: Maxsyn (E+, n = 13 and E−, n = 2), Alto (E+, n = 50 and E−, n = 3), Bronsyn (E+, n = 39 and E−, n = 7) and Trojan (E+, n = 85 and E−, n = 5).

**Table 5:** The number of misclassified sub-samples identified by the hierarchal model built to discriminate between endophyte presence (E+) or absence (E−) in cultivars of perennial ryegrass seeds. Misclassification rates vary from 0 to 11.3%.

Step (Rule)	Endophyte Status	Endophyte Status	No. of Times	Total Misclassified	Total Misclassified
	(Classification)	(Actual)	Misclassified	(per Endophyte Status)	(per Cultivar)
(a) Maxsyn	E−	E+	0 of 13	0.0%	
	E+	E−	0 of 2	0.0%	0.0%
(b) Bronsyn	E−	E+	3 of 39	7.7%	
	E+	E−	0 of 7	0.0%	6.5%
(c) Trojan	E−	E+	3 of 85	3.5%	
	E+	E−	1 of 5	20.0%	4.4%
(d) Alto	E−	E+	6 of 50	12.0%	
	E+	E−	0 of 3	0.0%	11.3%

The validation dataset was used to test the hierarchical model. The number of NIRS sub-samples (1 scan/sub-sample) included Maxsyn (E+, n = 13 and E−, n = 2), Alto (E+, n = 50 and E−, n = 3), Bronsyn (E+, n = 39 and E−, n = 7), and Trojan (E+, n = 85 and E−, n = 5).

**Table 6:** The number of misclassifications (individual sub-samples) resulting from each step of the hierarchical model, compared to the actual class, using the external validation dataset (n = 201).

Classification	Actual	Class Misclassified	Times Misclassified	% Misclassified	%Total Misclassified
Alto E−	Bronsyn E−	Cultivar	2	1.0%	
Alto E+	Maxsyn E+	Cultivar	1	0.5%	
Alto E+	Trojan E+	Cultivar	2	1.0%	
Bronsyn E+	Trojan E+	Cultivar	1	0.5%	
Maxsyn E+	Trojan E+	Cultivar	3	1.5%	4.5%
Alto E−	Alto E+	Endophyte status	5	2.5%	
Bronsyn E−	Bronsyn E+	Endophyte status	3	1.5%	
Trojan E−	Trojan E+	Endophyte status	3	1.5%	
Trojan E+	Trojan E−	Endophyte status	1	0.5%	6.0%
Alto E−	Bronsyn E+	Cultivar and Endophyte status	1	0.5%	
Bronsyn E−	Trojan E+	Cultivar and Endophyte status	1	0.5%	
Bronsyn E+	Trojan E−	Cultivar and Endophyte status	3	1.5%	
Unclassified	Alto E+	Cultivar and Endophyte status	1	0.5%	3.0%
**Total**			**27**	**13.0%**	

## Data Availability

Data is available from the authors upon reasonable request.

## Supplementary Materials

The following supporting information can be downloaded at https://www.mdpi.com/article/10.3390/s25041264/s1, Figure S1: Images of perennial ryegrass seeds; Table S1: Pre-processing methods applied to NIRS spectra for the optimization of PLS-DA models; Table S2: Model optimization output illustrating the calibration (Cal), cross validation (CV), and prediction (Pred) results for the discrimination of perennial ryegrass seed cultivars (Alto, Bronsyn, Maxsyn, and Trojan); Table S3: Model optimization output illustrating the calibration (Cal), cross validation (CV), and prediction (Pred) results for the discrimination of perennial ryegrass seeds with the presence or absence of an endophyte (E+ or E−).
