# Supplementary material for: Predicting Perennial Ryegrass Cultivars and the Presence of an Epichloë Endophyte in Seeds Using Near-Infrared Spectroscopy (NIRS)
Source: Sensors (Basel). 2025 Feb 19;25(4):1264. doi: 10.3390/s25041264 (PMC11860381; doi:10.3390/s25041264)
Supplement: Supplementary file 1 [file sensors-25-01264-s001.zip › sensors-3441934-supplementary.pdf]

sensors

Supplementary

Predicting Perennial Ryegrass Cultivars and the Presence of an *Epichloë* Endophyte in Seeds Using Near-Infrared Spectroscopy (NIRS)

Simone Vassiliadis [1], Kathryn M. Guthridge [1], Priyanka Reddy [1], Emma J. Ludlow [1], Inoka K. Hettiarachchige [1] and Simone J. Rochfort [1,2,*]

[1] Agriculture Victoria Research, Bundoora, VIC 3083, Australia; simone.vassiliadis@agriculture.vic.gov.au (S.V.); kathryn.guthridge@agriculture.vic.gov.au (K.M.G.); priyanka.reddy@agriculture.vic.gov.au (P.R.); emma.ludlow@agriculture.vic.gov.au (E.J.L.); inoka.hettiarachchige@agriculture.vic.gov.au (I.K.H.)
[2] School of Applied Systems Biology, La Trobe University, Bundoora, VIC 3083, Australia
* Correspondence: simone.rochfort@agriculture.vic.gov.au

Supplementary Figure S1: Images of perennial ryegrass seed

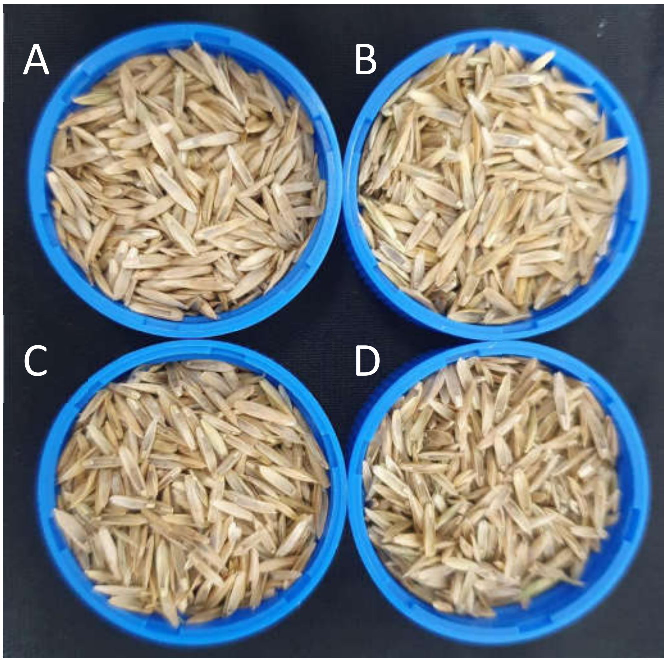

Figure S1. Perennial ryegrass seed A) Alto NEA12, B) Bronsyn NEA11, C) Maxsyn NEA21 and D) Trojan NEA2. Note. The blue contains are 3 cm in diameter.

Supplementary Table S1. Pre-processing methods applied to NIRS spectra for the optimization of PLS-DA models.

Model no.	Trend	Scatter correction	Spectral derivatives	Scaling and centering
1	Detrend	na	na	Mean Centre
2	Detrend	na	SavGol-1	Mean Centre
3	Detrend	na	SavGol-2	Mean Centre
4	Detrend	MSC (Mean)	SavGol-1	Mean Centre
5	Detrend	MSC (Mean)	SavGol-2	Mean Centre
6	Detrend	SNV	SavGol-1	Mean Centre
7	Detrend	SNV	SavGol-2	Mean Centre
8	Detrend	EMSC	SavGol-1	Mean Centre
9	Detrend	EMSC	SavGol-2	Mean Centre
10	na	SNV	SavGol-1	Mean Centre
11	na	SNV	SavGol-2	Mean Centre

MSC, Multiplicative Scatter/Signal Correction. SNV, Standard Normal Variate. EMSC, Extended Multiplicative Scatter/Signal Correction. SavGol-1, Savitsky-Golay first derivative with polynomial order 2 and 15 points in the smoothing window. SavGol-2, Savitsky-Golay second derivative with polynomial order 2 and 15 points in the smoothing window. na, not applicable.

Supplementary Table S2. Model optimization output illustrating the Calibration (Cal), cross validation (CV) and prediction (Pred) results for the discrimination of perennial ryegrass seed cultivars (Alto, Bronsyn, Maxsyn and Trojan).

Model no.	LVs	Cultivar	Sensitivity (Cal)	Class. Error (Cal)	Sensitivity (CV)	Class. Error (CV)	Sensitivity (Pred)	Class. Error (Pred)
1	8	Alto	0.90	9.5%	0.89	10.2%	0.93	9.2%
		Bronsyn	0.86	11.9%	0.84	13.2%	0.89	8.7%
		Maxsyn	0.96	6.4%	0.94	7.4%	0.83	11.2%

Model no.	LVs	Cultivar	Sensitivity (Cal)	Class. Error (Cal)	Sensitivity (CV)	Class. Error (CV)	Sensitivity (Pred)	Class. Error (Pred)
		Trojan	0.91	9.9%	0.91	10.4%	0.88	8.8%
2	5	Alto	0.89	12.7%	0.88	13.6%	0.87	13.4%
		Bronsyn	0.78	18.7%	0.77	19.8%	0.85	13.7%
		Maxsyn	0.90	13.7%	0.90	14.2%	0.92	12.4%
		Trojan	0.81	17.8%	0.79	18.6%	0.81	15.3%
3	8	Alto	0.97	3.8%	0.95	5.2%	0.98	4.0%
		Bronsyn	0.84	12.2%	0.84	12.3%	0.89	8.7%
		Maxsyn	1.00	1.7%	0.98	3.0%	1.00	1.3%
		Trojan	0.89	10.2%	0.87	10.9%	0.89	10.5%
4	7	Alto	0.98	5.1%	0.98	5.2%	0.93	7.2%
		Bronsyn	0.81	16.3%	0.80	17.3%	0.87	12.3%
		Maxsyn	0.98	3.8%	0.96	5.2%	1.00	2.1%
		Trojan	0.86	14.4%	0.84	16.2%	0.88	12.0%
5	8	Alto	0.98	2.0%	0.98	2.0%	0.96	3.0%
		Bronsyn	0.90	11.0%	0.89	11.0%	0.89	11.0%
		Maxsyn	1.00	1.0%	1.00	1.0%	1.00	1.0%
		Trojan	0.85	12.0%	0.85	13.0%	0.90	9.0%
6	7	Alto	0.97	5.9%	0.97	6.3%	0.93	8.5%
		Bronsyn	0.79	17.9%	0.79	18.1%	0.87	12.7%
		Maxsyn	0.98	3.2%	0.98	3.8%	1.00	2.1%
		Trojan	0.89	13.5%	0.87	15.3%	0.89	11.4%
7	5	Alto	0.87	17.2%	0.86	17.7%	0.83	17.3%
		Bronsyn	0.80	19.3%	0.80	19.2%	0.83	19.0%
		Maxsyn	0.88	11.8%	0.88	12.0%	1.00	5.0%
		Trojan	0.80	17.3%	0.79	17.5%	0.81	13.5%
8	8	Alto	0.98	3.6%	0.95	5.3%	0.96	4.3%
		Bronsyn	0.83	16.4%	0.80	18.6%	0.87	13.0%
		Maxsyn	0.98	2.7%	0.98	2.9%	0.92	6.0%
		Trojan	0.89	11.4%	0.87	12.6%	0.88	11.5%
9	7	Alto	0.99	2.9%	0.97	4.0%	0.98	2.6%
		Bronsyn	0.86	13.8%	0.84	15.7%	0.89	10.6%
		Maxsyn	1.00	1.0%	1.00	1.3%	1.00	0.5%
		Trojan	0.85	12.5%	0.83	14.0%	0.86	10.8%
10	7	Alto	0.96	6.2%	0.96	6.8%	0.93	8.2%
		Bronsyn	0.89	11.9%	0.88	12.7%	0.91	11.8%
		Maxsyn	0.98	3.4%	0.98	3.4%	1.00	1.9%
		Trojan	0.87	12.8%	0.83	14.7%	0.88	12.0%
11	7	Alto	0.99	3.5%	0.96	4.9%	0.96	3.6%
		Bronsyn	0.87	12.5%	0.85	14.2%	0.96	8.9%

Model no.	LVs	Cultivar	Sensitivity (Cal)	Class. Error (Cal)	Sensitivity (CV)	Class. Error (CV)	Sensitivity (Pred)	Class. Error (Pred)
		Maxsyn	1.00	0.7%	1.00	1.5%	1.00	0.5%
		Trojan	0.86	12.8%	0.85	13.7%	0.88	10.6%

Class. Error, classification error. LVs, latent variables. The calibration dataset included: Alto (n= 160), Bronsyn (n= 146), Maxsyn (n= 48) and Trojan (n= 252). The validation dataset included: Alto (n= 53), Bronsyn (n= 46), Maxsyn (n= 12) and Trojan (n= 90).

Supplementary Table S3. Model optimization output illustrating the Calibration (Cal), cross validation (CV) and prediction (Pred) results for the discrimination of perennial ryegrass seed with the presence or absence of endophyte (E+ or E-).

Model no.	LVs	Endophyte Presence	Sensitivity (cal)	Class. Error (cal)	Sensitivity (CV)	Class. Error (CV)	Sensitivity (pred)	Class. Error (pred)
1	3	E+	0.70	24.9%	0.70	25.0%	0.67	20.0%
		E-	0.80	24.9%	0.80	25.0%	0.93	20.0%
2	6	E+	0.80	17.1%	0.79	18.8%	0.84	11.1%
		E-	0.86	17.1%	0.84	18.8%	0.93	11.1%
3	4	E+	0.79	24.5%	0.78	25.6%	0.81	19.4%
		E-	0.72	24.5%	0.71	25.6%	0.80	19.4%
4	5	E+	0.72	18.6%	0.71	24.1%	0.72	17.6%
		E-	0.91	18.6%	0.81	24.1%	0.93	17.6%
5	4	E+	0.72	23.4%	0.72	26.3%	0.74	22.9%
		E-	0.81	23.4%	0.76	26.3%	0.80	22.9%
6	9	E+	0.88	12.0%	0.86	14.6%	0.89	12.3%
		E-	0.89	12.0%	0.85	14.6%	0.87	12.3%
7	4	E+	0.72	22.9%	0.71	26.4%	0.74	23.2%
		E-	0.82	22.9%	0.76	26.4%	0.80	23.2%
8	6	E+	0.77	16.2%	0.76	20.1%	0.77	18.0%
		E-	0.91	16.2%	0.84	20.1%	0.87	18.0%
9	4	E+	0.72	22.9%	0.71	26.4%	0.74	23.2%
		E-	0.82	22.9%	0.76	26.4%	0.80	23.2%
10	3	E+	0.64	27.4%	0.63	28.4%	0.63	21.6%
		E-	0.81	27.4%	0.80	28.4%	0.93	21.6%
11	2	E+	0.50	29.7%	0.48	30.4%	0.45	30.8%
		E-	0.91	29.7%	0.91	30.4%	0.93	30.8%

Class. Error, classification error. LVs, latent variables. The calibration dataset included: E+ (n= 727) and the validation dataset included: E- (n= 186).
